# PVT1 Promotes Cancer Progression via MicroRNAs

**DOI:** 10.3389/fonc.2019.00609

**Published:** 2019-07-15

**Authors:** Wenxi Wang, Ruoyu Zhou, Yuwei Wu, Yicong Liu, Wenjia Su, Wei Xiong, Zhaoyang Zeng

**Affiliations:** ^1^NHC Key Laboratory of Carcinogenesis and Hunan Key Laboratory of Translational Radiation Oncology, Hunan Cancer Hospital and The Affiliated Cancer Hospital of Xiangya School of Medicine, Central South University, Changsha, China; ^2^The Key Laboratory of Carcinogenesis and Cancer Invasion of the Chinese Ministry of Education, Cancer Research Institute, Central South University, Changsha, China; ^3^Hunan Key Laboratory of Nonresolving Inflammation and Cancer, Disease Genome Research Center, The Third Xiangya Hospital, Central South University, Changsha, China

**Keywords:** PVT1, miRNA, cancer, sponge, splicing

## Abstract

Non-coding RNA (ncRNA) plays a regulatory role in a variety of cellular activities. And long non-coding RNA (lncRNA) is one of the important kinds of ncRNA. Previous studies have shown that various lncRNAs are involved in the progression of cancer. LncRNA plasmacytoma variant translocation 1 (PVT1) is a newly discovered oncogenic factor that has been confirmed to be overexpressed in many cancer cells. Moreover, the role of PVT1 in cancer development is closely linked to microRNAs (miRNAs). PVT1 can act as a “sponge” for miRNAs to inhibit their activities, thereby affecting proliferation, invasion, and angiogenesis of cancer. In addition, PVT1 itself can be spliced and processed into several miRNAs such as miR-1204 and miR-1207, which can also regulate the development of cancer. This review summarizes various pathways through which PVT1 regulates the progression of cancer via miRNAs. We also propose additional regulatory mechanisms of PVT1 and their potential clinical applications.

## Introduction

Cancer is a non-communicable disease which threatens human health, with a global death toll ranking second only to cardiovascular disease (1). An increasing number of studies have shown that non-coding RNA (ncRNA) plays an important regulatory role in the development of cancer and participates in various cellular processes, such as DNA replication, RNA transcription, and protein synthesis, transport, and degradation. There are many types of ncRNA identified in cells, of which long non-coding RNA (lncRNA) and miRNA catch most of the attention.

PVT1 is an important oncogenic lncRNA highly expressed in cancer cells. The human *PVT1* gene is located in 8q24, which is widely recognized as a cancer-associated region (2). The carcinogenic effect of PVT1 has been confirmed in various tumors, such as gallbladder cancer (3), non-small-cell lung cancer (4–6), colon cancer (7, 8), leukemia (9, 10), hepatocellular cancer (11–13), breast cancer (14), and ovarian cancer (15). Multiple miRNA response elements are found on PVT1, to which specific miRNAs can bind and such that these miRNAs are silenced and the expression of certain proteins are upregulated, which ultimately affects the proliferation, invasion, and drug resistance of tumor cells. This mechanism is called the miRNA-mediated sponge interactions (MMI) effect. Currently, researches show that there are more than 20 miRNAs that can be sponged by PVT1, including miR-30a, miR-128, miR-186 etc. (16–18). In addition, PVT1 itself can also be spliced into 6 different miRNAs, namely miR-1204, miR-1205, miR-1206, miR-1207-5p, miR-1207-3p, and miR-1208, with either cancer-inducing or cancer-inhibiting function (19, 20). These discoveries point to a direction for the study of PVT1 and tumor development.

This review systematically outlines the manners through which PVT1 affects the development of the tumor via miRNAs. We propose a regulatory network that centered on PVT1, analyze the feasibility of using PVT1 as a tumor molecular marker and discuss its potential clinical applications.

## PVT1 Regulates Tumor Progression Through Sponging miRNAs

### PVT1 Affects Tumor Proliferation

Abnormal proliferation of tumor cells is an important feature that distinguishes tumor tissues from normal tissues. It is characterized by changes in the cell cycle, inhibition of apoptosis, and abnormality in energy metabolism. Several studies have confirmed that *PVT1*, as a potential oncogene, can promote tumor proliferation.

**Figure 1:**
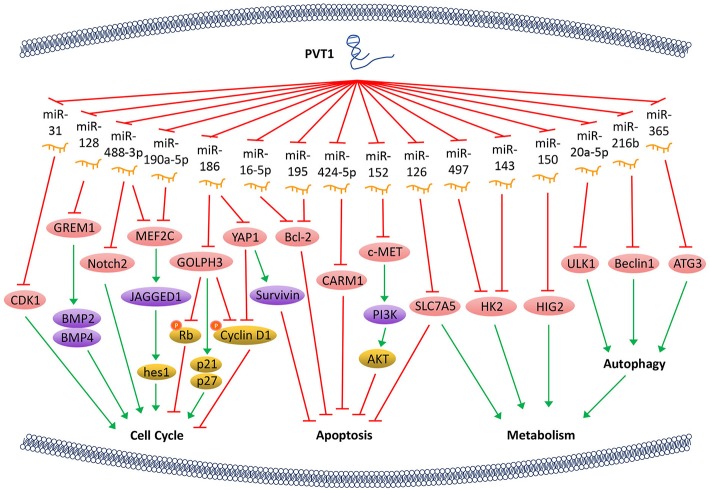
PVT1 regulates tumor progression. LncRNA PVT1, acting as a miRNA sponge, regulates the cell cycle, apoptosis, and energy metabolism of cancer cells through a variety of pathways, thus promotes tumor proliferation.

Bone morphogenetic protein (BMP) is an important member of the TGF-β superfamily and influences many important biological processes such as tumor proliferation by regulating a series of downstream genes. PVT1 can counteract the inhibitive expression of gremlin 1 (GREM1) through sponging miR-128, thereby affecting the downstream proteins BMP2 and BMP4-mediated signaling pathway, thus maintaining the proliferative activity of tumor cells (21). Notch signaling pathway affects multiple processes of normal morphogenesis, including differentiation of pluripotent progenitor cells, cell proliferation, cell boundary formation, and apoptosis. PVT1/miR-190a-5p/miR-488-3p/MEF2C/JAGGED1 pathways were shown to be involved in the promotion of tumor cell proliferation. By binding to miR-190a-5p/miR-488-3p, PVT1 promotes the overexpression of myocyte enhancer factor 2C (MEF2C), which is a direct downstream target of miR-190a-5p and miR-488-3p. MEF2C, in turn, upregulates the expression of JAGGED1 via enhancing its promoter activity (22). As a ligand of the Notch signaling pathway, JAGGED1 promotes the expression of its downstream genes such as *hes1*, to regulate tumor cell proliferation. In addition, PVT1 regulates the expression of Notch2 through acting on miR-488-3p (23), thereby halts the cell cycle at the G0/G1 phase. PVT1 can also regulate the expression of Golgi phosphoprotein 3 (GOLPH3) by sponging miR-186 and increase the expression of p21 and p27, and thus decreasing the phosphorylation of cyclin D1 and Rb, which ultimately shortens the cell cycle (24). Furthermore, PVT1 downregulates miR-31 to enhance the expression of cyclin-dependent kinases 1 (CDK1) and facilitates tumor cell proliferation, migration, and invasion (25) (Figure 1).

The Hippo-YAP pathway is involved in regulating tumorigenesis. PVT1 activates the transcriptional activator yes-associated protein 1 (YAP1) (18) via inactivating miR-186, thereby allows YAP1 to enter the nucleus and increase the expression of its downstream gene *Survivin* and the cell cycle protein D1. Ultimately, PVT1 shortens the cell cycle and inhibits cell apoptosis. Bcl-2 is a key factor that alters the permeability of the mitochondrial outer membrane and allows tumor cells to escape apoptosis. With PVT1 overexpression, miR-16-5p (26) or miR-195 (27) are inhibited, and thus their downstream gene *Bcl-2* is overexpressed. PVT1 also plays a role in regulating the apoptosis of tumor cells through activating c-MET/PI3K/AKT and co-activator-associated arginine methyltransferase 1 (CARM1) signaling pathways by sponging miR-152 and miR-424-5p, respectively (5, 28) (Figure 1).

The PVT1/miR-497/HK2 axis which regulates tumor cell energy metabolism is another important pathway that promotes tumor cell proliferation. The overexpression of PVT1 inhibits miR-497 and restores the activity of hexokinase 2 (HK2), thereby increasing the consumption of glucose and the production of lactic acid, which promotes cell proliferation (29). PVT1 also modulates HK2 expression by competitively binding to endogenous miR-143 in tumor cells, which promotes cell proliferation and metastasis by regulating aerobic glucose metabolism (3). In addition, PVT1 acts as a sponge for miR-126 and increases the expression of energy metabolism-related enzyme SLC7A5 in mitochondria, which is another important mechanism through which PVT1 enhances energy metabolism and promotes tumor cell proliferation (30). Hypoxia-inducible protein 2 (HIG2) is found to be a target molecule of miR-150. PVT1 knockdown inhibits the expression of HIG2 via up-regulating the expression of miR-150, and thus ultimately suppresses the tumorigenesis (13). It has also been shown that PVT1 plays an important role in autophagy in tumor cells. As a sponge of miR-20a-5p, PVT1 can increase the expression of unc-51-like kinase 1 (ULK1), which promotes autophagy in tumor cells, thereby providing sufficient energy for tumor growth (31). Beclin1, an important component of PI3K complex, helps locate autophagy proteins to the autophagic vacuoles after being phosphorylated by ULK1. Hence, PVT1 can regulate autophagy via PVT1/miR-216b/Beclin-1 axis (32). Another investigation reveals that PVT1 can upregulate autophagy-related gene 3 (ATG3) expression via acting as an endogenous sponge for miR-365 (12) (Figure 1).

### PVT1 Induces Tumor Metastasis

The evaluation of the invasive and metastatic ability of cancer cells is a key indicator of cancer staging and prognosis. PVT1 has been shown to be involved in the following steps during the epithelial-mesenchymal transition (EMT) and distant metastasis of cancer cells: (1) alteration of the adhesion between tumor cells and the surrounding tissues, and influence on cell detachment from the primary focus; (2) degradation of the extracellular matrix; (3) enhancement of cancer cell motility via modification of cytoskeleton; and (4) promotion of angiogenesis in tumor tissues (33).

**Figure 2:**
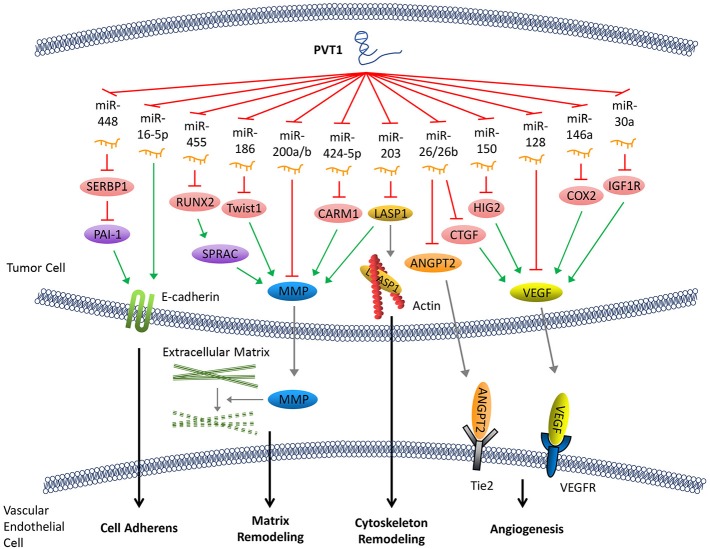
PVT1 promotes tumor metastasis. By sponging miRNAs, PVT1 downregulates these miRNAs and results in reduced expression of E-cadherin, enhanced production of MMP and angiogenesis factors, and remodeling of the cytoskeleton.

Cadherin is an important intercellular adhesive molecule, and its decreased expression can promote the EMT of cancer cells. PVT1 regulates plasminogen activator inhibitor 1 RNA-binding protein (SERBP1), plasminogen activator inhibitor-1 (PAI-1), and other molecules by competitively binding to miR-448, thereby reducing the expression of E-cadherin and promoting the invasion of cancer cells (34–36). In addition, E-cadherin can also be down-regulated directly by PVT1 through regulation of miR-16-5p (26, 37) (Figure 2).

Following abscission from the surrounding tissues, tumor cells secrete matrix metalloproteinase (MMP), which promotes the decomposition of the surrounding matrix. The expression of MMP can be upregulated by several signaling pathways that are regulated by PVT1. Both miR-455 and miR-30d-5p can be sponged by PVT1 (8, 38). These two pathways form regulatory loops with runt-related transcription factor 2 (RUNX2), leading to the upregulation of secreted protein acid rich in cysteine (SPARC), which ultimately enhances the expression of MMP (39). Similarly, PVT1 can also upregulate MMP9 through PVT1/miR-150/HIG2 pathway (40), PVT1/miR-424-5p/CARM1 pathway (5), or by binding to miR-200a and miR-200b (41). Moreover, the discovery of the regulatory axis of PVT1/miR-186/Twist1 (42) confirmed that PVT1, through its sponging function, promotes the expression of Twist1 which is a transcription factor related to EMT, thereby promoting the EMT (Figure 2).

Remodeling of the cytoskeleton system promotes invasion and metastasis of cancer cells. PVT1 can sponge miR-203, thereby increasing the expression of Lim and SH3 domain protein 1 (LASP1) which is an actin-binding protein that binds to actin and alters its structure (43). It has been shown that an increase in LASP1 expression can promote the transformation of the cytoskeleton, rendering it more suitable for tumor cell invasion and metastasis (44). LASP1 also participates in other signaling pathways and ultimately upregulates the expression of MMP (45) (Figure 2).

Tumor angiogenesis not only facilitates the transport of nutrients required for cell growth but also creates conditions that allow for distant metastasis through blood vessels. Angiogenesis in tumor tissues is regulated by angiogenesis factors and angiogenesis inhibitors. PVT1 elevates the expression of various angiogenesis factors by binding to their corresponding regulatory miRNAs. For example, PVT1 can elevate the expression of cyclooxygenase-2 (COX2) mRNA through binding with miR-146a. COX2 can initiate the synthesis of the vascular endothelial growth factor (VEGF) family proteins, which are among the most potent factors regulating angiogenesis (46). The PVT1/miR-150/HIG2 (40) pathway also enhances the expression of VEGF. Meanwhile, PVT1 can boost the expression of insulin-like growth factor 1R (IGF1R), which in turn promotes the expression of VEGF by sponging miR-30a (16). In addition, it has been shown that miR-128 directly targets the 3'-UTR of vascular endothelial growth factor C (VEGFC). PVT1, a sponge for miR-128, eliminates the inhibitory effect of miR-128 on the expression of VEGFC, which facilitates its expression (47). Furthermore, other reports have demonstrated that PVT1 binds to and enhances the degradation of miR-26 or miR-26b, thus upregulates the expression of connective tissue growth factor (CTGF) and angiopoietin 2 (ANGPT2), both of which are important angiogenic factors (7, 48) (Figure 2).

## PVT1 Regulates Tumor Progression Through Encoding miRNAs

**Figure 3:**
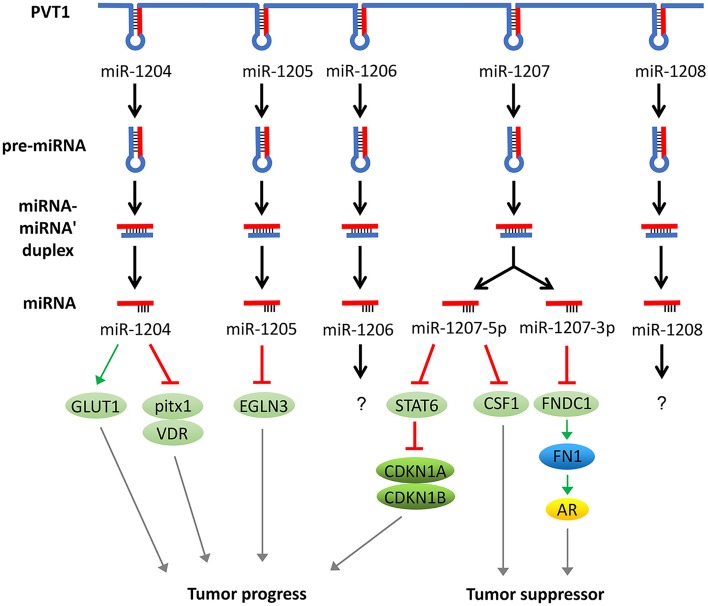
PVT1 regulates tumor progression through encoded miRNAs. PVT1 itself can be trimmed and processed into several miRNAs, known as miR-1204, 1205, 1206, 1207-3p, 1207-5p, and 1208. Among these miRNAs, miR-1204, 1205, 1207-3p, and 1207-5p have been shown to promote or inhibit cancer, while the effects of the remaining two miRNAs in cancer remain unknown.

In addition to acting as a molecular sponge for miRNAs, PVT1 itself can also be trimmed and processed into several miRNAs (miR-1204, 1205, 1206, 1207-3p, 1207-5p, 1208). These miRNAs can also regulate the development of tumors. For example, overexpression of miR-1207-5p reduces the expression of signal transducers and activators of transcription (STAT6), thereby activates cyclin-dependent kinase inhibitor 1A (CDKN1A) and CDKN1B to regulate the cell cycle and promote tumor cell proliferation (49). In addition, the elevated expression of miR-1204 not only significantly increases glucose transporters 1 (GLUT-1) expression and glucose uptake but also suppresses the expression of pitx1 and Vitamin D (1,25- dihydroxy vitamin D3) receptor (VDR), which ultimately promotes cell proliferation, invasion, and metastasis (50, 51). In addition, miR-1205 downregulates the expression of the Egl-9 family hypoxia-inducible factor 3 (EGLN3) and promotes cell proliferation and cell cycle progression and inhibits hydrogen peroxide-induced apoptosis (52). Surprisingly, miR-1204, miR-1207-3p, and miR-1207-5p have exhibited an inhibitory function of tumor progression in some studies. For instance, miR-1204 and miR-1207 are shown to enhance the sensitivity of tumor cells to chemotherapeutic drugs (53, 54). In another report, the miR-1207-3p/FNDC1/FN1/AR pathway is shown to be involved in the inhibition of tumor proliferation and migration, and induction of apoptosis (55). Furthermore, the miR-1207-5p/CSF1 axis can also inhibit tumor proliferation and migration by regulating tumor microenvironment (56). These contradictory tumor-suppressing effects caused by the inhibition of PVT1 through miR-1204, miR-1207-3p, and miR-1207-5p warrant further investigation (57) (Figure 3).

## Prospects

Existing studies have shown that PVT1 is overexpressed in a variety of tumors, and its expression is closely associated with tumor proliferation and apoptosis, invasion and metastasis, angiogenesis, and drug resistance. A recent study shows that knock-down of *PVT1* can increase the radiosensitivity of the tumor (5), suggesting its role as an oncogene to promote tumor progression. The role of PVT1 in tumor is closely associated with a variety of miRNAs and their downstream pathways. In addition to the sponge-like effect mentioned above, PVT1 can also affect miRNAs through other mechanisms, thereby promoting tumor progression. A previous study showed that PVT1 can increase the expression of miR-214 by enhancing the binding of enhancer of zeste homolog 2 (EZH2) to the miR-214 promoter, which ultimately promotes tumor cell proliferation and invasion (58). In addition, PVT1 also downregulates the expression of miR-146a by increasing the activity of DNA methylase, which induces the methylation of the CpG island in miR-146a precursor, thereby affecting the growth of tumor cells (59). At the same time, with the discovery of PVT1-encoded miRNAs, an increasing number of studies have revealed that these miRNAs also participate in the regulation of tumor progression. Currently, there are several studies involving PVT1-encoded miR-1204, miR-1205 and miR-1207. However, not all studies show that these miRNAs promote tumor development. Additionally, whether miR-1206 and miR-1208 plays specific roles in cancer remains unknown. Overall, the effects of PVT1 on tumors are closely associated with miRNA regulation.

In addition to its long-chain form, PVT1 also exists in a circular form. The circular PVT1 (circPVT1) locus is contained within the lncPVT1, which originates from the exon 2 of the *PVT1* gene. Several studies have demonstrated that circPVT1 is also abnormally expressed in tumor cells. Lorena Verduci et al. showed that the mutant p53/YAP/TEAD transcription complex enhanced the expression of circPVT1, which in turn acted as an oncogene to regulate tumor proliferation by affecting the expression of miR-497-5p (60). In addition, circPVT1 also promotes drug resistance of tumor cells (61) and interacts with miRNAs as a competing endogenous RNA (ceRNA) just like lncPVT1. Hence, not only lncPVT1 but also circPVT1 is closely linked to tumor development. There have been several studies exploring lncPVT1's clinical application. The results reveal that lncPVT1 is a potential biomarker for some tumors as its expression is abnormal and the detecting technology has been optimized (62–66). And as a more stable form, circPVT1 may be more valuable in clinic practice. In summary, PVT1 will be used for tumor screening, malignant and prognosis evaluating, or even as a molecule target for cancer treatment in the near future.

Certain mechanisms through which PVT1 affects tumor development remain unclear. It has been noticed that *PVT1* and the oncogene *c-MYC* coexist in the same chromosomal region, namely the 8q24 region. They are coamplified (67), and c-MYC can regulate the expression of PVT1. They both can promote tumor proliferation. Moreover, Salehi et al. demonstrated that the expression of the *c-MYC* gene was downregulated when *PVT1* was knocked out, and thus the apoptosis and necrosis of cancer cells increased (68). This suggests that PVT1 and c-MYC are related to tumorigenesis and mutually regulate each other. A recent report also proposed the mutual regulatory relationship between PVT1 and MYC. In addition to the oncogenic lncPVT1, the PVT1 promoter affects tumor development by affecting the transcription of *PVT1* and *c-MYC*, and functions independently of lncPVT1 (69). Whether there are more oncogenes or tumor suppressors within the 8q24 region and what are the interrelationships among them warrant future explorations, which will greatly facilitate our fundamental understanding of cancer development.

## Author Contributions

All authors listed have made a substantial, direct and intellectual contribution to the work, and approved it for publication.

### Conflict of Interest Statement

The authors declare that the research was conducted in the absence of any commercial or financial relationships that could be construed as a potential conflict of interest.

## Abbreviations

ATG3: autophagy-related gene 3
ANGPT2: angiopoietin 2
AR: androgen receptor
BMP: bone morphogenetic protein
CDK1: cyclin-dependent kinases 1
CARM1: co-activator-associated arginine methyltransferase 1
c-Met: cellular-mesenchymal to epithelial transition factor
COX2: cyclooxygenase-2
CTGF: connective tissue growth factor
CDKN1: cyclin-dependent kinase inhibitor 1
CpG island: 5 ’-C-phosphate-G-3’ island
circPVT1: circular PVT1
ceRNA: competing endogenous RNA
EMT: epithelial-mesenchymal transition
EGLN3: Egl-9 family hypoxia-inducible factor 3
EZH2: enhancer of zeste homolog 2
FNDC1: fibronectin type III domain containing 1
FN1: fibronectin 1
GREM1: gremlin 1
GOLPH3: Golgi phosphoprotein 3
GLUT-1: glucose transporters 1
HK2: hexokinase 2
HIG2: hypoxia-inducible protein 2
IGF1R: insulin-like growth factor 1R
lncRNA: long-chain non-coding RNA
LASP1: Lim and SH3 domain protein
miRNA: microRNA
MMI: miRNA-mediated sponge interactions
MEF2C: myocyte enhancer factor 2C
MMP: matrix metalloproteinase
ncRNA: non-coding RNA
PVT1: plasmacytoma variant translocation 1
PI3K: phosphatidylinositol 3-kinase
PAI-1: plasminogen activator inhibitor-1
RUNX2: runt-related transcription factor 2
SERBP1: plasminogen activator inhibitor 1 RNA-binding protein
SPARC: secreted protein acid rich in cysteine
STAT: signal transducers and activators of transcription
TEAD: TEA domain family member 1
ULK1: unc-51-like kinase 1
VEGF: vascular endothelial growth factor
VDR: Vitamin D (1,25- dihydroxy vitamin D3) receptor
YAP1: yes-associated protein 1.
